# Bibliometric analysis of ChatGPT in medicine

**DOI:** 10.1186/s12245-024-00624-2

**Published:** 2024-04-04

**Authors:** Sharanya Gande, Murdoc Gould, Latha Ganti

**Affiliations:** 1 Academy of the Lakes, Land O’ Lakes, FL USA; 2 Rollins College, Winter Park, FL USA; 3 University of Central Florida, Orlando, FL USA; 4 Warren Alpert Medical School of Brown University, RI Providence, USA

## Abstract

**Introduction:**

The emergence of artificial intelligence (AI) chat programs has opened two distinct paths, one enhancing interaction and another potentially replacing personal understanding. Ethical and legal concerns arise due to the rapid development of these programs. This paper investigates academic discussions on AI in medicine, analyzing the context, frequency, and reasons behind these conversations.

**Methods:**

The study collected data from the Web of Science database on articles containing the keyword “ChatGPT” published from January to September 2023, resulting in 786 medically related journal articles. The inclusion criteria were peer-reviewed articles in English related to medicine.

**Results:**

The United States led in publications (38.1%), followed by India (15.5%) and China (7.0%). Keywords such as “patient” (16.7%), “research” (12%), and “performance” (10.6%) were prevalent. The Cureus Journal of Medical Science (11.8%) had the most publications, followed by the Annals of Biomedical Engineering (8.3%). August 2023 had the highest number of publications (29.3%), with significant growth between February to March and April to May. Medical General Internal (21.0%) was the most common category, followed by Surgery (15.4%) and Radiology (7.9%).

**Discussion:**

The prominence of India in ChatGPT research, despite lower research funding, indicates the platform’s popularity and highlights the importance of monitoring its use for potential medical misinformation. China’s interest in ChatGPT research suggests a focus on Natural Language Processing (NLP) AI applications, despite public bans on the platform. Cureus’ success in publishing ChatGPT articles can be attributed to its open-access, rapid publication model. The study identifies research trends in plastic surgery, radiology, and obstetric gynecology, emphasizing the need for ethical considerations and reliability assessments in the application of ChatGPT in medical practice.

**Conclusion:**

ChatGPT’s presence in medical literature is growing rapidly across various specialties, but concerns related to safety, privacy, and accuracy persist. More research is needed to assess its suitability for patient care and implications for non-medical use. Skepticism and thorough review of research are essential, as current studies may face retraction as more information emerges.

## Introduction

The emergence of AI chat programs presents two paths: one in which it is used to enhance and optimize the way we interact with queries and problems, becoming a spark that ushers in a new era of rapid academic and technological development, and another in which it is used to replace the need for one’s personal understanding. Additionally, it poses a multitude of ethical considerations and has even created certain legal gray areas, indicative of its development surpassing the speed of societies. This paper will serve as a means to better understand the context of how academic circles, countries, and institutions are discussing AI within the realm of medicine, how much they are discussing it, and conjecturing into why these conversations are occurring using supporting research.

ChatGPT is a Natural Language Processing (NLP) AI software that generates responses to any query a user may input [1]. It provides quick and clear responses and, as a result, is used widely in the same capacity as a search engine but with a more dynamic ability to interpret complicated questions, compile relevant information, and respond. Holders of professional titles such as PhDs are predicted to be affected by this; they may be at risk of decreasing importance due to AI’s ability to generate the same accurate and precise reports, curtailing the novelty of such research [2]. 

Although only existed for less than a year among the public, ChatGPT has made a significant impact on higher education and a variety of academic disciplines, including medicine. ChatGPT’s potential use in medicine arises from its success in aiding with diagnosis and decision-making due to its efficiency, timeliness, and access to a vast wealth of research and information. This allows it to compare medical knowledge between institutions globally, enhance communication among patients and hospital workers, and even assist in answering questions, whether they be medical queries, dosing information, or even medical exams [2, 3]. . Another recent use of the platform, which has been considered to simplify the process of medical writing, is its ability to extract medical information and perform searches to create research drafts [1].

## Methods

The data set collected was obtained using an advanced search on the Web of Science database for the keyword “ChatGPT,” not case sensitive, resulting in 1440 articles published from January 1st, 2023 to September 30th, 2023. Web of Science was used because of its unique positioning as an interdisciplinary hub for global research boasting its representation of over 256 disciplines and 15 million researchers, and its ability to portray a general climate of research. The Web of Science database also allows filtering by index and category, which was performed to only allow articles from the Science Citation Index Expanded (SCI-EXPANDED) and Emerging Sources Citation (ESCI) due to their medical focus. The categories were also limited to those pertaining to the medical field; all others were excluded. Analysis was performed on the remaining 786 medically related journal articles containing the keyword “ChatGPT”.

The criterion for inclusion was that the articles be peer-reviewed, pertaining to the medical field, within the data range analyzed, and in the English language. The title, abstract, and keywords of the included studies all included “ChatGPT”.

The Web of Science also allows for data collection of bibliometric information, which can later be imputed into specialized software for interpretation. This interpretation was performed using VOSviewer 1.6.19, a software specialized in analyzing articles bibliometrically producing visual and analytic findings for speculation. The metadata acquired was then examined using 4 different categorical distinctions -- country of origin, journal, month of publication, and keywords -- and mapped using the same software.

## Results

**Fig. 1:**
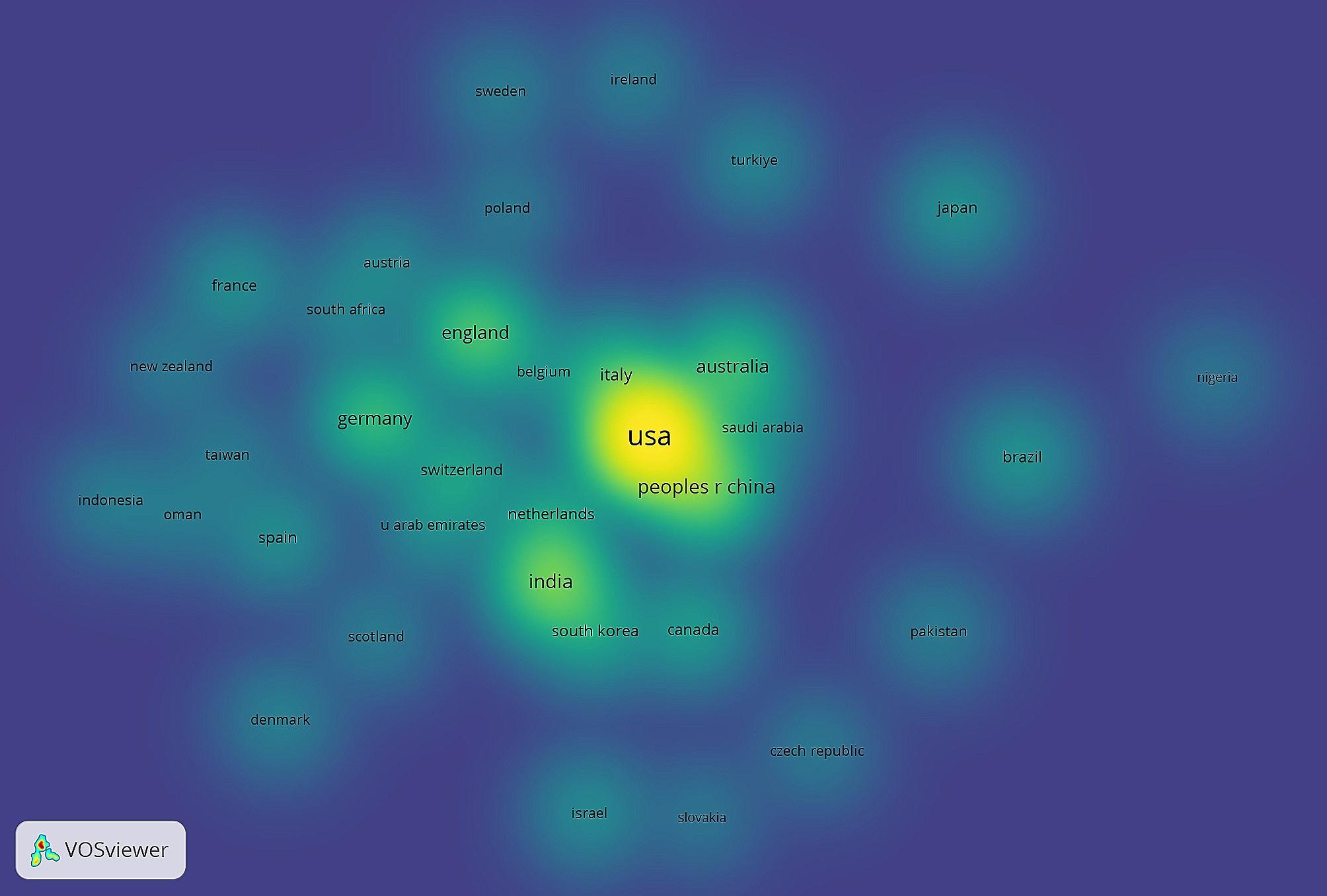
Geographical collaboration network heat map of publications Jan - Sep 2023

**Fig. 2:**
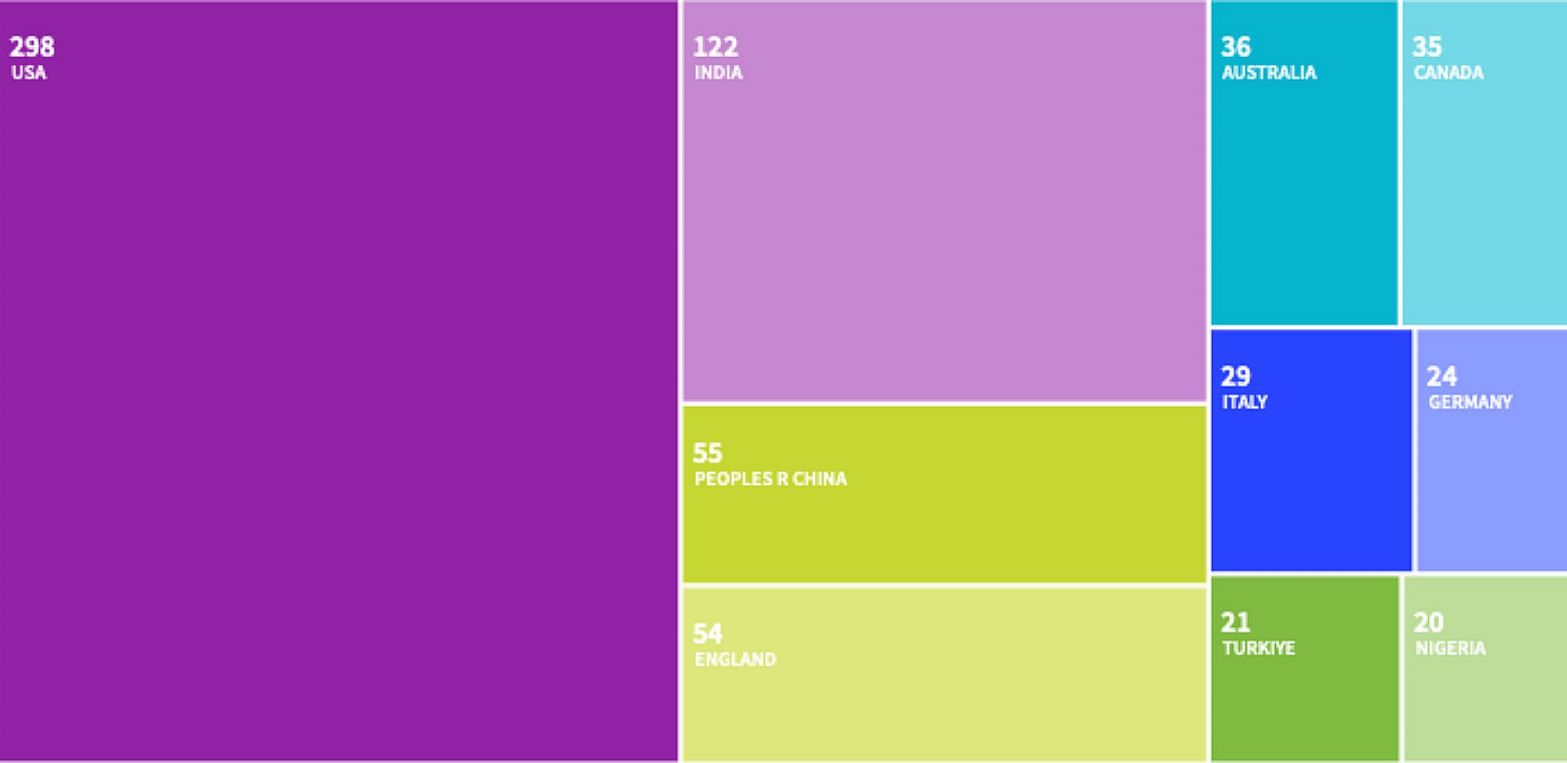
Treemap chart of geographic contributions to publications Jan - Sep 2023. The three most prevalent keywords that appear are patient(16.7%), research(12%), and performance(10.6%). Other topically relevant keywords were medical education(4.5%), ethic(1.8%), and plagiarism(1.3%)

There were 786 documents retrieved from the Web of Science Core Collection, after filtering for medical-related research that used the term ChatGPT in 2023. The country leading in total number of publications was the US at 38.1% [Fig. 1]. Following the US was India and the People’s Republic of China at 15.5% and 7.0% respectively. England and Australia were the fourth and fifth top contributors with 7.5% and 6.5% of total publications [Fig. 2].

**Fig. 3:**
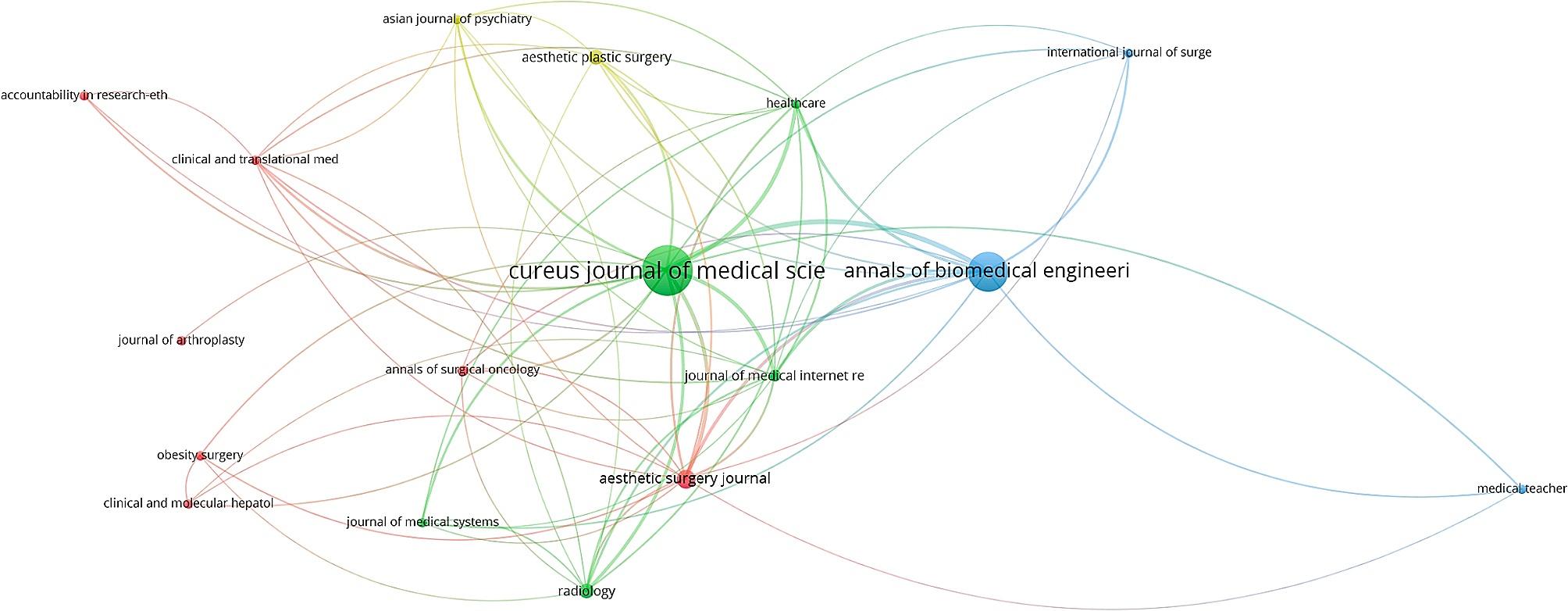
Publication bibliographic coupling by journals Jan - Sep 2023

The majority of articles analyzed were published in the Cureus Journal of Medical Science(11.8%) [Fig. 3]. Annals of Biomedical Engineering followed with the second most publications(8.3%). The third most published journal, Aesthetic Surgery Journal, is significantly less prolific contributing to 2.3% of all the documents analyzed, 72.3% fewer articles than the Annals of Biomedical Engineering. Both the Aesthetic Plastic Journal and the Radiology Journal have published 1.4% percent of all the articles retrieved, tying them as the fourth most substantial journal on ChatGPT. All remaining journals contained eight or fewer(≤ 1.0%) articles on the topic of ChatGPT.

**Fig. 4:**
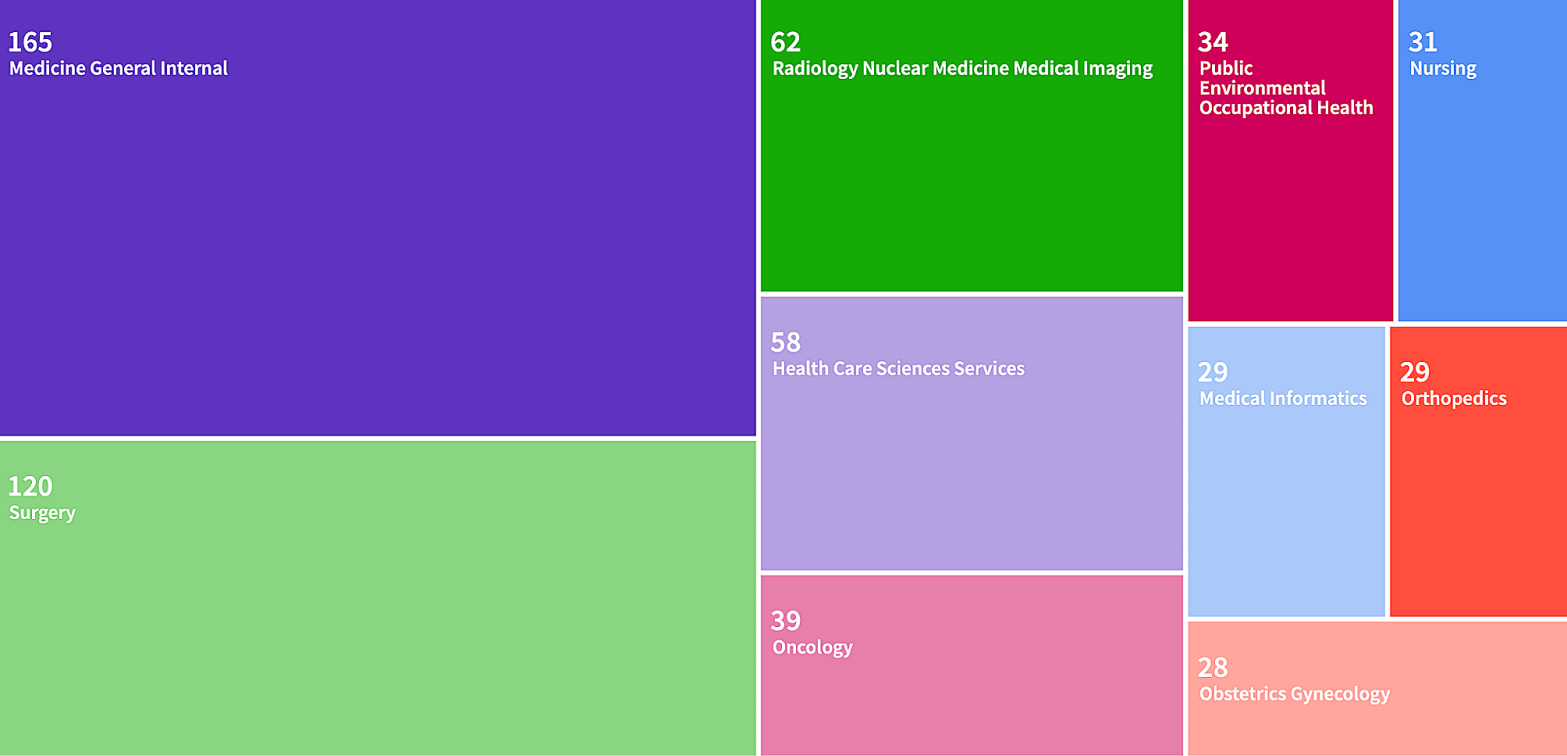
Web of Science Categories Treemap chart of publications Jan - Sep 2023

When indexing the articles by month, January 2023 had the least publications(≤ 0.005%) while August 2023, the latest month included in data collection, had the most(29.3%) [Fig. 4]. Of growth, from February to March and from April to May experienced the largest in terms of percentage with 1700% and 134%. The month of August exhibited the highest increase of papers published with 104 more papers than in July, increasing the rate of publication to 83%. June was the only month that did not experience research growth, declining 10% from the previous month.

The Web of Science categories are determined by up to 6 tags associated with any given journal. Web of Science designates its tags using subjects of the journal, author and editorial affiliations, funding, citations, and other elements such as a journal’s bibliographic categorization in other databases and a journal’s sponsors. The category of Medicine General Internal includes the most articles included in the analysis(21.0%), followed by Surgery(15.4%), Radiology(7.9%), and Health Care Science Services (7.8%). All remaining categories contained less than 5% of the journals included in this study.

The 5 most cited articles were also collected for their merit in understanding academic discussions surrounding ChatGPT. The most cited article (121 citations) was “ChatGPT utility in healthcare education, research, and practice: Systematic review on the promising perspectives and valid concerns,” published in March 2023 [4]. “ChatGPT and other large language models are double-edged swords” and “ChatGPT: the future of discharge summaries” were the second most referenced articles, with 90 citations each [5, 6]. “ChatGPT and the future of medical writing” had 84 citations, and “Nonhuman “authors” and implications for the integrity of scientific publications and medical knowledge” had 77 citations [7, 8](Biswas, 2023; Flanagin et al., 2023).

## Discussion

### Countries

Analyzing the three leading countries in publications regarding ChatGPT yields results that deviate from the norm of global research efforts. The leading country in publications, the United States (US), is often considered the highest researching entity due to spending more on research than any other country and offers the least notable finding [9]. India historically has low research funding, though it is improving, which draws particular attention to their dominance in ChatGPT research [10, 11]. Elucidating this finding is India’s status as the second most avid country using ChatGPT, accounting for 8.5% of the total traffic [11]. Many concerns with ChatGPT’s use and accuracy in medicine go beyond clinical settings and focus on how public use of the platform could lead to medical misinformation. For this reason, the popularity of the platform publicly and privately throughout the country, being mirrored by the country’s research institutions and funding efforts, is crucial in managing potential medical misuse of ChatGPT without explicit medical supervision.

The People’s Republic of China, which is the third most publishing country on ChatGPT with 9.8%, does not allow public access to the platform [12]. With major universities in the country also passing explicit bans on the platform, findings suggest that ChatGPT is researched abstractly on ethical grounds or with special permissions. Furthermore, these findings suggest that the interest lies not with ChatGPT as a program, but with NLP AI and its applications more generally. Additionally, funding for research in China is set to eclipse the US and tension can be seen with competition for NLP AI research superiority [9, 13, 14]. Further research on the nature of ChatGPT research in China specifically is needed, but findings thus far appear to demonstrate that national bans on the platform do not affect publication outputs.

### Journals

Cureus is an open-access, peer-reviewed, general medical journal and currently is the most prolific in its publication of articles surrounding ChatGPT. Cureus was one of the first journals to issue a call for papers specifically using ChatGPT, which likely has contributed to its numbers. While Cureus is not technologically specified - as compared to the second most published journal in ChatGPT, Annals of Biomedical Engineering - its dominance in the space can be attributed to its new age structure and business model. Of note, Cureus was one of the first journals to issue a call for papers specifically using ChatGPT, which likely has contributed to its numbers. Cureus’ success in publishing articles on ChatGPT may also hinge on its ability to peer-review articles and publish submitted work quickly [15]. As such, it participates in “rapid research,” or the practice of publishing articles to appropriately respond to the void of information on what was previously an under-researched topic. What was observed in “rapid research” during the beginning of the COVID-19 pandemic is that while the speed of publication of articles was 11.5 times faster than publications on influenza, the rate of retractions and withdrawals was also significantly higher [16–18].

### Medical disciplines

The Aesthetic Surgery Journal and Aesthetic Plastic Journal were two of the most prolific in publishing articles on ChatGPT and surgery was the second largest category, raising questions about ChatGPT’s usage in healthcare fields such as plastic surgery. A bibliometric study that addresses a plastic surgeon’s use of ChatGPT specifically yields four key findings: use in research and creation of original work, clinical applications, surgical education, and ethics/commentary on previous studies [19]. These findings are reinforced by our study, particularly in regard to the prevalence of the keywords medical education, research, and ethics. The mirroring of these interests fortifies the claim that ChatGPT has significant merit in medical education, with evidence to support that it is being examined for use in surgery but that ethical considerations remain a concern.

Literature surrounding radiology also demonstrates its use in innovative procedures and potential use for mitigating physician workloads [20]. Our study shows that it was the third most published category, suggesting it is particularly applicable to innovations in radiology, and similar studies support this claim [21]. ChatGPT is able to learn as it is fed more data and additionally excels at image analysis and pattern recognition. With physician burnout plaguing the healthcare industry, ChatGPTs use in automating such tasks serves as a potential solution [22]. AI is still subject to error however, and requires careful review if it is to be used in such a way, as to ensure patient safety, demonstrating a need for large in-depth studies into the platform’s reliability as a means of physician automation [20].

On May 24th, 2023 a bibliometric analysis of ChatGPT in Obstetrics and Gynecology (OBGYN) during a 69-day period found 0 relevant articles on its application [23]. Our study’s findings show a significant increase with 28 articles categorized under obstetric gynecology reflecting ChatGPTs adoption by more disciplines. Additionally, the disciplines of oncology, nursing, and medical informatics are all represented significantly in the top 10 categories of ChatGPT medical research. ChatGPT and NLP AI’s uses are extraordinarily dynamic; as more research is being done on its accuracy generally in medicine, more disciplines have begun incorporating it practically.

A similar study to this was conducted by Barrington et al. in Sep of 2023. The findings are mirrored by our results even with the addition of a wider date range of data collection. This demonstrates a trend in the direction of ChatGPT’s medical research and also solidifies the need for unresolved gaps in research to be addressed, namely into the limitations of ChatGPT ethically and in regards to accuracy and safety [24].

### Limitations

Among the limitations of this study is its reliance on the Web of Science as the sole source of data. While Web of Science is expansive, there are discrepancies between it and a database such as PubMed, particularly in newer articles [25]. Additionally, this study only examined the keyword ChatGPT and did not explicitly include other forms of NLP AI limiting its ability to create a general image of this technology in medicine.

As with all medical research, academic interests and concerns are bound to change with the addition of new articles. Especially in the case of ChatGPT given the rapid research that surrounds it and its position as an avant-garde tool, even weeks after the publication of this article can see a reshuffling in research priorities. Despite this fact, the study provided does encompass the largest bibliometric date range on ChatGPT as of this time.

## Conclusion

The literature on ChatGPT in medicine is extensive considering how new the platform is. Many medical specialties are exploring applications of the platform, and this study has shown that month over month an increasing number of disciplines are getting involved. Much of this research shares the same limitations, the safety, privacy, and accuracy of using ChatGPT for patient care. This gap in the literature needs further research if proposed applications are to be put into practice. Our analysis also emphasizes much-needed skepticism in reviewing said research, as much of the current studies could be at risk for retraction as more information is found. Concerns around the use of ChatGPT’s use medically in nonclinical settings are also found, a topic that is sorely underrepresented in current findings. The conclusion of this paper necessitates that more research be done into ChatGPT’s reliability for providing appropriate patient care in order to allow for applications in clinical settings, and the implications of ChatGPT’s use in non-medically trained hands.

## Data Availability

No datasets were generated or analysed during the current study.
